# MEDAG enhances breast cancer progression and reduces epirubicin sensitivity through the AKT/AMPK/mTOR pathway

**DOI:** 10.1038/s41419-020-03340-w

**Published:** 2021-01-18

**Authors:** Zhiyu Li, Chenyuan Li, Qi Wu, Yi Tu, Changhua Wang, Xin Yu, Bei Li, Zhong Wang, Si Sun, Shengrong Sun

**Affiliations:** 1 Department of Breast & Thyroid Surgery, Renmin Hospital of Wuhan University, Wuhan, Hubei People’s Republic of China; 2 Department of Pathophysiology, Wuhan University School of Basic Medical Sciences, Wuhan, Hubei People’s Republic of China; 3 Department of Pathology, Renmin Hospital of Wuhan University, Wuhan, Hubei People’s Republic of China; 4 Department of Clinical Laboratory, Renmin Hospital of Wuhan University, Wuhan, Hubei People’s Republic of China

**Keywords:** Breast cancer, Chemotherapy, Oncogenes

## Abstract

Breast cancer (BC) is the most common malignancy among women. Mesenteric estrogen-dependent adipogenesis gene (MEDAG) was first reported as a novel adipogenic gene, and its involvement and mechanism in visceral adiposity were analyzed. However, the role of MEDAG in BC is unclear. The biological roles and corresponding mechanisms were investigated in vitro and in vivo. We found that MEDAG was highly expressed in BC samples and that a high MEDAG expression was correlated with clinicopathological characteristics and poor survival in BC patients. MEDAG knockdown inhibited cell proliferation, invasion, and migration; triggered epithelial-to-mesenchymal transition (EMT); and enhanced epirubicin sensitivity in vitro. The opposite results were observed in MEDAG-overexpressing cells. The inhibition of MEDAG suppressed tumor growth and metastasis in vivo. Mechanistically, MEDAG knockdown led to decreased phosphorylation levels of AKT, increased levels of p-AMPK, and reduced levels of p-mTOR, while the overexpression of MEDAG had the opposite effects. Moreover, the activation of p-AKT and inhibition of p-AMPK restored the effect of MEDAG on EMT and chemosensitivity in BC cell lines, indicating that MEDAG functions as an oncogene by regulating the AKT/AMPK/mTOR pathway. MEDAG regulates BC progression and EMT via the AKT/AMPK/mTOR pathway and reduces chemosensitivity in BC cells. Therefore, MEDAG is a promising target for BC.

## Introduction

Breast cancer is the most common cancer among women. Over the past few decades, the incidence rate of breast cancer has slightly increased since 2004^1^, and the mortality rate has decreased since 1989^2^, which may reflect important advances in the detection and treatment of breast cancer. Despite this progress, breast cancer remains the second leading cause of cancer-related death among women primarily due to tumor progression and therapeutic resistance.

The epithelial-to-mesenchymal transition (EMT) is characterized by polarized epithelial cells converting to motile mesenchymal cells in the embryonic process. Profound changes in cell motility and morphology enable cells to travel long distances and are involved in internal organ formation^3^. Moreover, EMT is highly involved in tumor progression and confers aggressive traits, such as migration, invasion, metastatic potential, and drug resistance, to cancer cells^4^.

Mesenteric estrogen-dependent adipogenesis gene (MEDAG), also known as Meda-4, was first reported as a novel adipogenic gene involved in visceral adiposity. A previous study showed that MEDAG overexpression enhanced preadipocyte differentiation, lipid accumulation, and glucose uptake in adipocytes^5^. In addition, other studies showed that MEDAG was upregulated in canine meningioma and osteoarthritis^6,7^. A recent report showed that MEDAG was associated with adverse clinical features and a poor prognosis among patients with papillary thyroid microcarcinoma^8^. However, knowledge regarding the function of MEDAG in breast cancer is limited.

Here, we demonstrate for the first time that MEDAG plays a role in breast cancer and extensively investigated the effects of MEDAG in vitro and in vivo. In this study, we elucidate the expression of MEDAG and its association with prognosis. Furthermore, we reveal the functions of MEDAG in breast cancer progression and EMT by conducting cell-based studies and analyses of mouse models and human breast cancer samples. Additionally, we provide evidence of the effect of MEDAG on chemosensitivity in breast cancer cells. Finally, the mechanism of MEDAG in tumor progression and chemosensitivity was investigated. Taken together, our study examined the potential role and mechanism of MEDAG in breast cancer progression and chemosensitivity.

## Materials and methods

### Patient samples and immunohistochemistry

A cohort of 101 human breast cancer paraffin-embedded samples and 10 normal breast tissues were collected from the Renmin Hospital of Wuhan University. The diagnoses were confirmed by histopathology, and the detailed clinicopathological features were obtained from the clinical records and pathology reports. Follow-up results of at least 5 years were used. All human participants involved in this study provided informed consent, and all methods were approved by the Institutional Ethics Committee of the Renmin Hospital of Wuhan University. The immunohistochemistry (IHC) staining and evaluation were performed as previously described^9^. The major steps in this process included deparaffinization, antigen retrieval, blocking, incubation with primary antibodies, washing, incubation with secondary antibodies, washing, DAB staining, washing, mounting, and observation. The following antibodies were used for the IHC analysis: anti-MEDAG (Biorbyt, orb380371), E-Cadherin (Cell Signaling Technology, #14472), N-Cadherin (Cell Signaling Technology, #13116), and Ki67 (Santa Cruz, sc-23900).

### Bioinformatics analysis

The mRNA profiling data of breast cancer patients were obtained from The Cancer Genome Atlas (TCGA) data portal (https://tcga-data.nci.nih.gov/tcga/). The differentially expressed genes (DEGs) between the high MEDAG expression group and the low MEDAG expression group were analyzed by R software. Gene ontology (GO) annotates the biological process, molecular function and cellular components of genes, and the Kyoto Encyclopedia of Genes and Genomes (KEGG) was used for the analyses of genomes, biological pathways, diseases, and drugs. The Database for Annotation, Visualization and Integrated Discovery (DAVID, https://david.ncifcrf.gov/) was utilized to obtain the GO annotations and KEGG pathways of the DEGs. Moreover, ImageGP (http://www.ehbio.com/ImageGP/index.php/Home/Index/index.html) was used to visualize the enrichment outcomes. The correlations between MEDAG and the EMT-related markers were calculated by GEPIA (http://gepia2.cancer-pku.cn/#index). The predicted protein models were estimated by I-TASSER (https://zhanglab.ccmb.med.umich.edu/I-TASSER).

### Cell line culture

Human breast cancer cell lines, including MCF-7 and MDA-MB-468, were obtained from the American Type Culture Collection (ATCC, Manassas VA, USA) and incubated according to the corresponding methods. Epirubicin was purchased from Pfizer Pharmaceutical Co. Ltd. (Wuxi, China) and dissolved in physiological saline. MK2206 (p-AKT inhibitor), SC79 (p-AKT agonist) and Compound C (p-AMPK inhibitor) were purchased from Selleck (Shanghai, China) and dissolved in DMSO.

### Gene transfection and virus infection

For the siRNA transfections, the cells were transfected with MEDAG siRNA (5′-GCAGUUUCUCUGACCGAAATT-3′) or scramble siRNA (5′-UUCUCCGAACGUGUCACGUTT-3′) (GenePharma, China) by iMAX (Invitrogen, USA). The plasmid containing the full-length human MEDAG gene was constructed by GeneChem (Shanghai, China). For the plasmid transfections, the cells were transfected using Lipofectamine 3000 (Invitrogen, USA) according to the manufacturer’s instructions.

The human MEDAG-targeting shRNA and control shRNA lentiviral plasmids were purchased from GeneChem (Shanghai, China). Stable knockdown cell lines were generated by lentivirus infection and selected with puromycin.

### Western blot analysis

The cells were lysed, and the proteins were extracted in RIPA lysis buffer with protease inhibitors and phosphorylase inhibitors on ice. The proteins were separated using SDS-PAGE and transferred to NC membranes. The membranes were blocked in 5% nonfat milk, incubated with primary antibodies overnight at 4 °C, and subsequently incubated with secondary antibodies for 1 h at room temperature. The results were visualized by an Odyssey Infrared Imager (Li-COR Biosciences, USA). The relevant antibodies are listed in Supplementary Table 1.

### Cellular proliferation, invasion and migration assays

For the measurement of cell proliferation, a Cell Counting Kit-8 (CCK-8) assay was used according to the manufacturer’s instructions (CK04, Dojindo, Japan). Transwell assays with Matrigel (plain DMEM:Matrigel = 9:1) were performed to measure cell invasion. Equal amounts of cells were seeded in the upper chambers (Corning, USA) in serum-free medium, and DMEM with 10% FBS was added to the lower chambers. After 24 h, the invasive cells were fixed and stained with 0.1% crystal violet.

Wound-healing assays were used to verify cell migration. A cell monolayer was spread on a plate, and then, a micropipette tip was used to wound the monolayer. Photographs were taken 0 and 48 h after wounding under a microscope (Olympus, Japan).

### Flow cytometry

Cell apoptosis was verified by a FITC Annexin V Apoptosis Detection Kit (556547, BD Pharmingen, USA). The cells were seeded on a plate, treated with 5 μmol/l epirubicin for 12 h, and then processed following the manufacturer’s protocol. The results were determined by a FACScan flow cytometer (FACScan, Becton Dickinson).

### qRT-PCR

The total RNA was extracted from the cells and tissues by TRIzol reagent, and reverse transcription was performed with a TransScript First-Stand cDNA Synthesis Kit (TaKaRa, Japan). qRT-PCR was carried out on an ABI-7900HT Real-Time PCR System using SYBR Green MasterMix (TaKaRa, Japan). The following primer sequences were used: MEDAG-F: aggacgtacgcgtttcttgt; MEDAG-R: gaattactgagcccgaacca. GAPDH-F: gagtccactggcgtcttca; and GAPDH-R: ggggtgctaagcagttggt.

### Animal experiments

All animal experiments were performed in accordance with the Declaration of Helsinki and were approved by the Animal Ethics Committee of Wuhan University. For the proliferation assay, MDA-MB-468 cells stably expressing control shRNA (shCtrl) or MEDAG shRNA were generated. Four-week-old female BALB/c nude mice were randomly assigned to the following two groups: control (injected with shCtrl cells) and knockdown (injected with MEDAG shRNA cells). The cells (2.5 × 10^6^ cells per mouse) were inoculated subcutaneously into the right iliac fossa of the mice. After 6 weeks, the mice were sacrificed, and the xenografts were removed for analysis.

For the metastasis assay, luciferase-expressing MDA-MB-231-Luc cells were also divided into the control (injected with shCtrl-Luc cells) and knockdown (injected with MEDAG shRNA-Luc cells) groups, and the cells were injected into the fourth mammary fat pads of the mice (1 × 10^6^ cells per mouse). After the orthotopic tumors were observed, the mice were analyzed by noninvasive bioluminescence imaging using an IVIS Lumina II imaging system (Caliper Life Science, Hopkinton, MA).

### Statistical analysis

The statistical analysis was performed using GraphPad 8.0 and SPSS 20.0 software. The association between MEDAG and clinicopathological characteristics of the breast cancer patients was calculated by Pearson χ^2^ test or Fisher’s exact test. The survival outcomes were evaluated by the Kaplan-Meier method and log-rank test. The correlations between MEDAG and EMT markers in the tissue were calculated by Pearson’s or Spearman’s correlation coefficients. The multiple group comparisons were performed by one-way ANOVA. The data are representative of at least three independent experiments and are presented as mean ± S.D. *P* < 0.05 was considered statistically significant.

## Results

### High MEDAG expression is correlated with adverse clinicopathological characteristics and poor survival in breast cancer patients

**Fig. 1:**
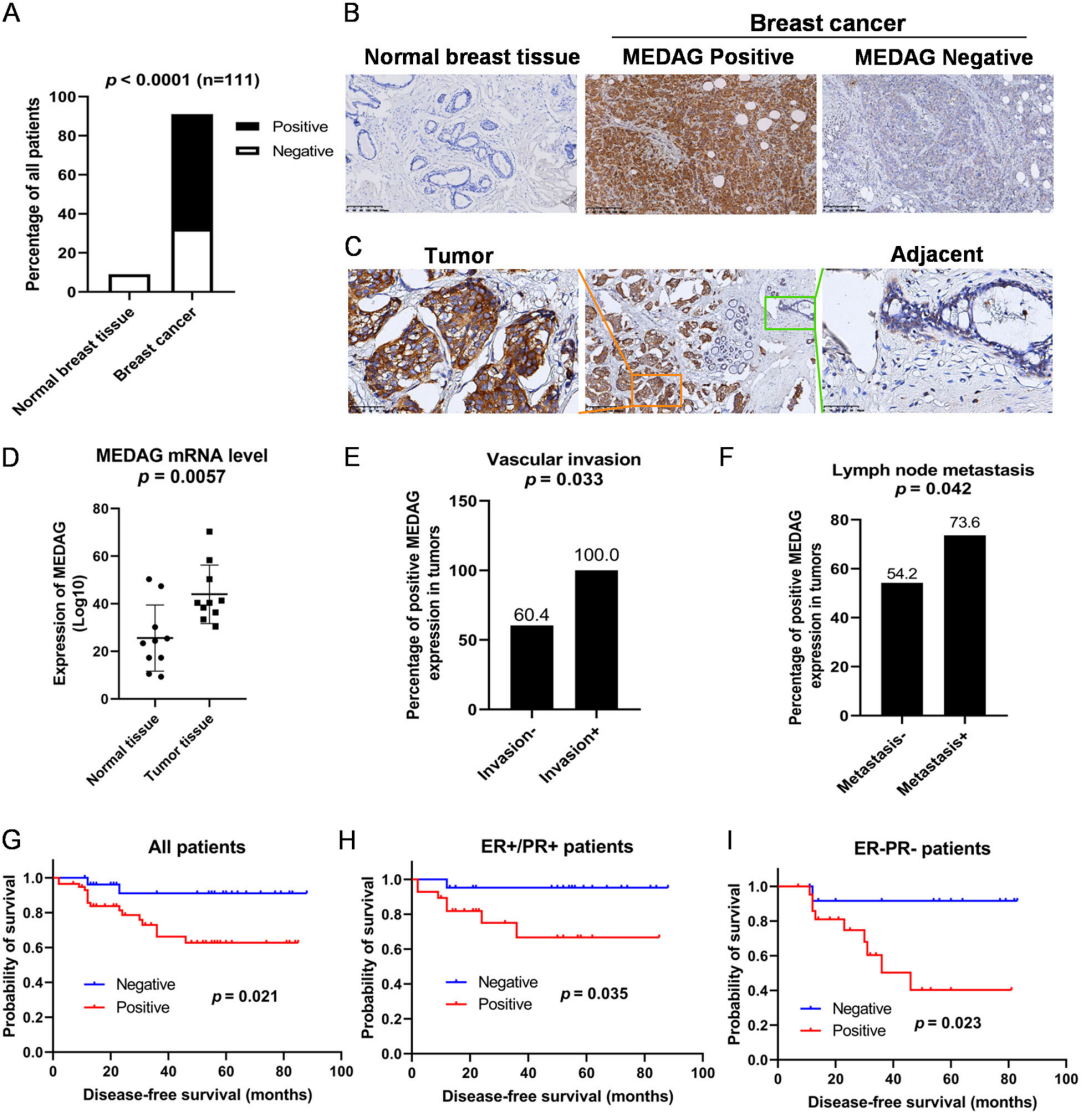
Elevated MEDAG expression indicates adverse clinicopathological characteristics and poor survival in breast cancer patients. **A**–**C** MEDAG expression in breast cancer samples and normal breast tissues was detected by an immunohistochemistry (IHC) assay. Quantitative analysis is shown in (**A**). Representative images are shown in (**B**, **C**) (magnification 200x). **D** mRNA levels of MEDAG in tumor tissues and corresponding normal tissues. **E**, **F** Percentages of human breast cancer samples with a high expression of MEDAG with a vascular invasion status and lymph node metastasis (χ^2^ test). **G**–**I** High MEDAG expression is correlated with a poorer disease-free survival in all breast cancer patients, ER+/PR+ patients and ER−/PR− patients (log-rank test).

To verify the clinical relevance of MEDAG in breast cancer, we examined MEDAG expression in 101 breast cancer tissues and 10 normal breast tissues. Fig. 1A, B shows that compared to the normal breast tissues, MEDAG was highly expressed in the breast cancer tissues, and MEDAG was overexpressed in the tumors but rarely expressed in the adjacent normal tissues in one sample (Fig. 1C). Moreover, we determined the mRNA level of MEDAG in 10 paired breast cancer and normal tissues. Fig. 1D shows that the mRNA level in the breast cancer tissues was higher than that in the normal tissues.

Then, we determined the associations between MEDAG expression and clinicopathological characteristics (Table 1) and found that MEDAG expression was significantly increased in the patients with vascular invasion compared with that in the patients without vascular invasion (*P* = 0.033, Fig. 1E). The patients with lymph node metastasis also had a significantly higher percentage of MEDAG positivity than those without metastasis (*P* = 0.042, Fig. 1F).

**Table 1:** Clinicopathological associations of MEDAG expression in breast cancer.

Variables	Negative, *n* = 36	Positive, *n* = 65	*P* value^*^
Age at diagnosis, years			0.775
<50	15 (41.7)	29 (44.6)	
≥50	21 (58.3)	36 (55.4)	
Menopausal status			0.603
Premenopausal	18 (50.0)	29 (44.6)	
Postmenopausal	18 (50.0)	36 (55.4)	
Tumor size(mm)			0.987
≤20	16 (44.4)	29 (44.6)	
>20	20 (55.6)	36 (55.4)	
Lymph node metastasis			**0.042**
Negative	22 (61.1)	26 (40.0)	
Positive	14 (38.9)	39 (60.0)	
Vascular invasion			**0.033**
Negative	36 (100.0)	55 (84.6)	
Positive	0 (0.0)	10 (15.4)	
ER			0.292
Negative	20 (55.6)	29 (44.6)	
Positive	16 (44.4)	36 (55.4)	
PR			0.307
Negative	21 (58.3)	34 (52.3)	
Positive	15 (41.7)	31 (47.7)	
HER2			0.096
Negative	26 (72.2)	36 (55.4)	
Positive	10 (27.8)	29 (44.6)	
Ki67			0.294
<4%	13 (36.1)	17 (26.2)	
≥14%	23 (63.9)	48 (73.8)	
Surgery			0.903
Mastectomy	34 (94.4)	61 (93.8)	
BCS	2 (5.6)	4 (6.2)	
Chemotherapy			0.461
None	7 (19.4)	9 (13.8)	
Yes	29 (80.6)	56 (86.2)	

^*^P values calculated by Pearson Chi squared testing; Bold if statistically significant, *P* < 0.05.

*ER* estrogen receptor, *PR* progesterone receptor, *HER2* human epithelial growth factor receptor-2, *BCS* breast-conserving surgery.

In addition, the survival outcomes were analyzed (Fig. 1G–I). The results demonstrated that the breast cancer patients negative for MEDAG had a better disease-free survival (DFS) than the patients positive for MEDAG (*P* = 0.021). In particular, the MEDAG-positive patients had a significant adverse outcome in both the ER+/PR+ subtype (*P* = 0.035) and the ER−/PR− subtype (*P* = 0.023).

### MEDAG promotes cell proliferation, invasion, and migration in vitro

First, we performed a GO analysis of MEDAG using R software and a DAVID analysis. The biological process results showed that the DEGs were particularly enriched in cell adhesion, positive regulation of cell proliferation, angiogenesis and migration, response to drug, immune/inflammatory response, aging, etc. (Fig. S1A). Moreover, the GO molecular function analysis showed that the DEGs were enriched in calcium ion binding, receptor activity, growth factor activity, etc. (Fig. S1B). In addition, three-dimensional protein models and the ligand binding sites were predicted by I-TASSER (https://zhanglab.ccmb.med.umich.edu/) (Fig. S2).

**Fig. 2:**
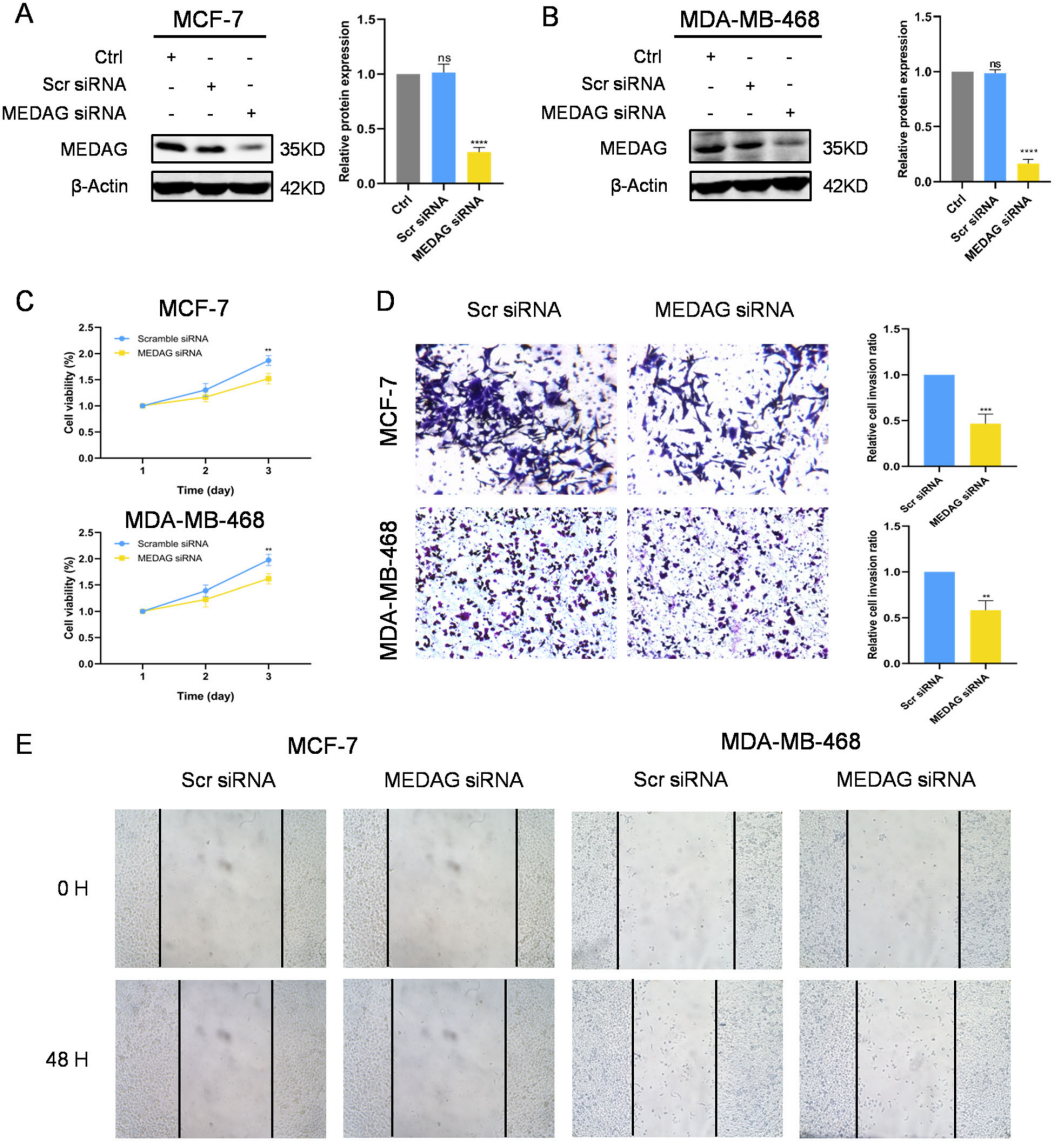
MEDAG knockdown inhibits cell proliferation and the pro-metastasis phenotype in breast cancer cells. **A**, **B** Knockdown efficiency of MEDAG in MCF-7 and MDA-MB-467 cell lines. **C** MEDAG knockdown inhibited breast cancer cell proliferation in MCF-7 (up) and MDA-MB-468 (down) cells. Cell proliferation was measured by a CCK-8 assay. **D** Transwell assays showing that MEDAG silencing decreased cell invasion in MCF-7 (up) and MDA-MB-468 (down) cells. Quantitative analysis of the invasion ratio is shown. **E** Wound-healing assay showing that MEDAG knockdown reduced cell migration. Representative images are shown at 0 and 48 h. The values represent mean ± SD of three independent experiments. **P < 0.01, ***P < 0.001, ****P < 0.0001 vs the control group.

To further demonstrate the role of MEDAG in breast cancer, we used siRNA to knockdown MEDAG and a plasmid to overexpress this gene in MCF-7 and MDA-MB-468 cells. In addition, CCK-8 assays, Transwell assays with Matrigel, and wound-healing assays were performed to confirm the function of MEDAG in breast cancer. The MEDAG protein level was similar between the control cells and the scramble siRNA cells (Fig. 2A, B). Compared with the control group and the scramble siRNA group, the group with MEDAG siRNA showed significantly decreased MEDAG expression in the two cell lines (Fig. 2A, B). The CCK-8 results showed that the MEDAG knockdown inhibited cell proliferation (Fig. 2C). As shown in Fig. 2D, silencing MEDAG dramatically reduced the relative cell invasion ratio compared with that in the control group. Moreover, silencing MEDAG strongly suppressed wound closure compared to that in the controls at 48 h.

Furthermore, we examined the effect of MEDAG overexpression in MCF-7 and MDA-MB-468 cells to strengthen our conclusion. As shown in Fig. S3, we transfected both cell lines with Flag-MEDAG and Flag-NC plasmids in the overexpression group and the control group (Fig. S3A, B). Compared to the control group, the overexpression group exhibited a marked increase in cell proliferation. Invasion and migration assays were also performed and showed that MEDAG overexpression enhanced the pro-metastatic phenotype in the breast cancer cell lines (Fig. S3D, E). Additionally, the rescue experiment showed that MEDAG overexpression increased cell proliferation and the pro-metastasis phenotype in the MEDAG^KD^ cells (Fig. S4).

The above results suggest that MEDAG promotes proliferation and pro-metastatic phenotypes, such as invasion and migration, in breast cancer cells.

### MEDAG triggers EMT in breast cancer

**Fig. 3:**
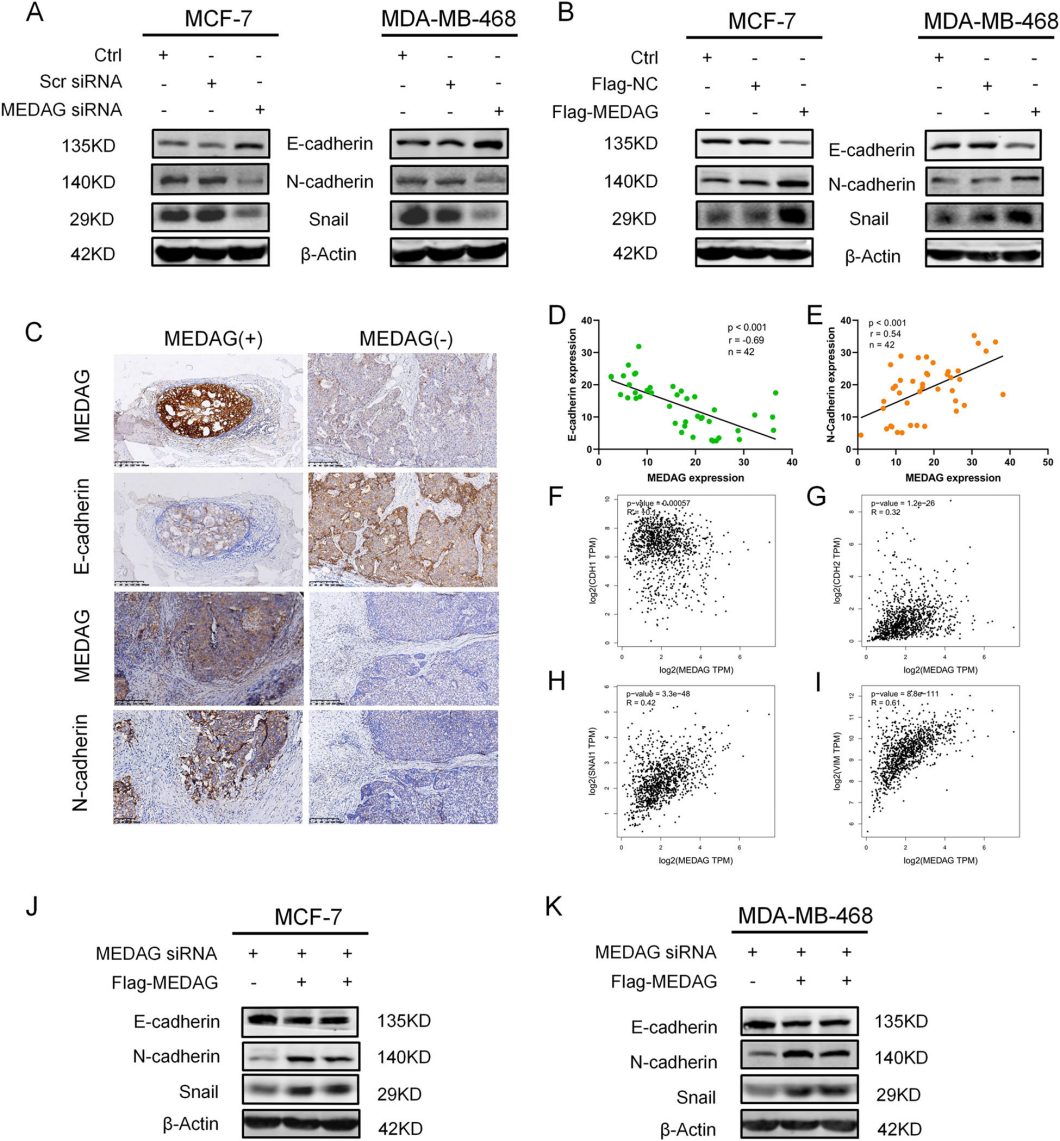
MEDAG triggers EMT in breast cancer cells. **A**, **B** Western blots of the EMT-related markers E-cadherin, N-cadherin, and Snail in MEDAG^KD^ cells and MEDAG^OE^ of MCF-7 and MDA-MB-468 cell lines. **C** Representative IHC staining images of MEDAG and EMT-related markers in breast cancer samples (magnification 200x). **D**, **E** Correlation analysis of protein expression between MEDAG and the EMT-related markers E-cadherin and N-cadherin in breast cancer samples by IHC. **F**–**I** Correlation analysis of the mRNA level between MEDAG and the EMT-related markers E-cadherin, N-cadherin, Snail and Vimentin in breast cancer from TCGA database. **J**, **K** MEDAG was overexpressed in MEDAG^KD^ cells, and the EMT-related proteins E-cadherin, N-cadherin and Snail were detected by western blot analysis. The correlation coefficients were calculated by Pearson’s or Spearman’s test.

To determine whether MEDAG contributes to EMT, we assessed EMT-related protein expression by western blot and IHC staining analyses. Deficiency of MEDAG led to an epithelial phenotype including an elevated expression of E-cadherin and a downregulated expression of N-cadherin and Snail in the two cell lines (Fig. 3A). In contrast, the overexpression of MEDAG resulted in a mesenchymal phenotype characterized by a decreased expression of E-cadherin and an upregulated expression of N-cadherin and Snail compared to that in the control group (Fig. 3B). Consistently, IHC staining of consecutive sections of breast cancer samples also supported these findings. In two consecutive sections of one sample, we observed that a high expression of MEDAG was associated with a low expression of E-cadherin but a high expression of N-cadherin, while a low expression of MEDAG had the opposite results (Fig. 3C). Moreover, we further examined the correlation between MEDAG and EMT proteins in a series of patients. The results showed that MEDAG expression was strongly negatively correlated with E-cadherin and positively correlated with N-cadherin (Fig. 3D, E). TCGA database was used to support the correlations between MEDAG and EMT markers at the mRNA level, and the correlations were consistent with the previous results (Fig. 3F–I). Intriguingly, we knocked down MEDAG, transfected Flag-MEDAG to rescue the MEDAG protein level, and tested whether MEDAG overexpression could reverse the changes in EMT. As shown in Fig. 3J, K, the rescue experiment partially restored the levels of N-cadherin and Snail and reversed the expression of E-cadherin. Therefore, our results show that MEDAG plays a crucial role in triggering EMT in breast cancer.

### Inhibition of MEDAG suppresses tumor growth and metastasis in vivo

**Fig. 4:**
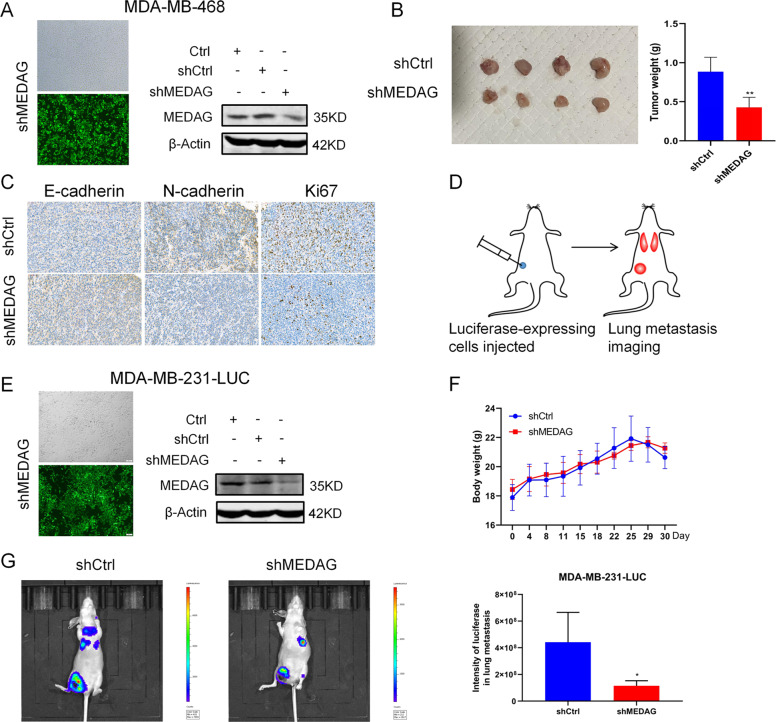
Inhibition of MEDAG suppresses tumor growth and metastasis in vivo. **A** Transfection efficiency of stable MEDAG knockdown in MDA-MB-468 cells (magnification 100x). **B** Comparison of tumor weights in various groups (*n* = 4). **C** Representative IHC staining of EMT-related markers and Ki67 in tumors in different groups. **D** Protocol of the metastasis experiment. **E** Transfection efficiency of stable MEDAG knockdown in MDA-MB-231-luc cells (magnification 100x). **F** Average weight of the mice in the two groups. **G** Lung metastasis was detected by noninvasive bioluminescence imaging in the two groups (*n* = 4).

Given the in vitro results, we then established in vivo xenograft models to verify the role of MEDAG in breast cancer growth and metastasis. First, MDA-MB-468 cells were infected with LV-shMEDAG or LV-shCtrl to assess tumor growth in the two groups. As shown in Fig. 4A, stable silenced cells were successfully established for the xenograft model in mice and subcutaneously inoculated into the right iliac fossa of the mice. After a period of time, we removed, photographed, and weigh the transplanted tumors to evaluate the growth of the xenografts. Compared with the corresponding control group, the tumor weight derived from the shMEDAG cells was significantly decreased (Fig. 4B). Representative IHC staining of different groups is shown in Fig. 4C. Then, luciferase-expressing control cells and luciferase-expressing shMEDAG cells were injected into the fourth mammary fat pad of mice to examine tumor metastasis (Fig. 4D). After a period of time, the mice were assessed by noninvasive bioluminescence imaging to detect lung metastasis. We monitored the average weight of the mice in the different groups and found no significant difference between the two groups (Fig. 4E). Moreover, the mice transplanted with the shCtrl cells had significantly greater and more bioluminescence in the lung than the mice in the shMEDAG group (Fig. 4F).

### MEDAG regulates breast cancer progression and EMT via the AKT/AMPK/mTOR pathway

**Fig. 5:**
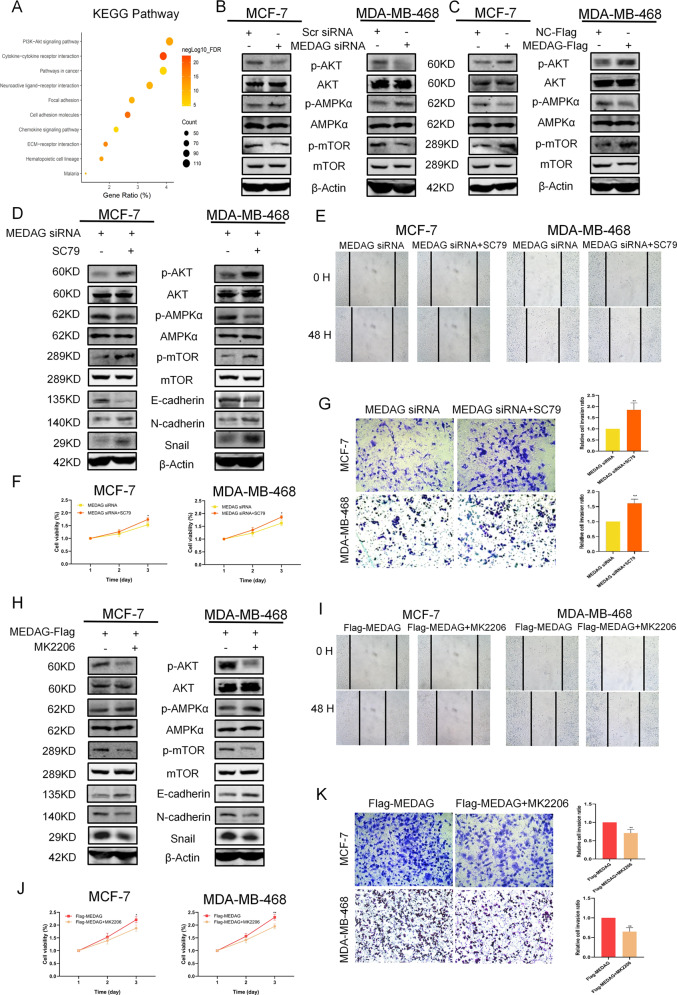
MEDAG regulates breast cancer progression and EMT via AKT signaling. **A** KEGG enrichment analysis of DEGs of MEDAG in breast cancer. **B**, **C** Western blot analysis of p-AKT, AKT, p-AMPK, AMPK, p-mTOR, and mTOR expression in the scramble group, MEDAG^KD^ cells, MEDAG^OE^ cells, and NC-FLAG group in MCF-7 and MDA-MB-468. **D**, **H** p-AKT, AKT, P-AMPK, AMPK, P-mTOR, mTOR and EMT-related proteins were detected in MEDAG^KD^ cells and MEDAG^KD^ cells treated with SC79 (10 µg/ml, 2 h) or MEDAG^OE^ cells and MEDAG^OE^ cells treated with MK2206 (2 µM, 18 h) by western blot analysis. **E**, **I** Wound-healing was used to examine the migration ability in the two cell lines treated as described above. **F**, **J** Cell growth was measured by CCK-8 in breast cancer cells treated as described above. **G**, **K** Transwell assay showing the cell invasion abilities in the groups treated as described above. Right: quantitative analysis of the invasion ratio is shown. The values represent mean ± SD of three independent experiments. **P < 0.01 vs the corresponding group.

To investigate the underlying mechanism of MEDAG in the regulation of the progression and EMT of breast cancer, we performed a KEGG analysis of the DEGs according to the MEDAG levels in breast cancer. The results showed that the most significantly enriched pathway was the PI3K/AKT pathway (*n* = 112, *P* = 3.02*10^-15^, FDR = 3.93*10^-12^, Fig. 5A). MEDAG was shown to be involved in glucose and lipid metabolism in adipocytes in a previous study^5^, and multiple studies have suggested that the AKT/AMPK/mTOR pathway may contribute to tumor development, energy metabolism, and protein synthesis in cancer; we finally identified the above pathway^10^.

The results of the MCF-7 and MDA-MB-468 cells showed that silencing MEDAG led to decreased phosphorylation levels of AKT, increased p-AMPK, and reduced p-mTOR, while MEDAG overexpression activated AKT/mTOR and inhibited AMPK activity (Fig. 5B, C). Notably, the treatment of the cells with SC79, which is an AKT agonist, could reactivate AKT signaling, reduce AMPK activity, and elevate the p-mTOR protein levels in the two types of MEDAG^KD^ cells; had an increased mesenchymal effect (N-cadherin, Snail); and decreased epithelial protein (E-cadherin) expression (Fig. 5D). Consistently, the experiments showed that AKT activation partially opposed the effect on cell proliferation, invasion, and migration in the MEDAG^KD^ cells (Fig. 5E–G). In contrast, MEDAG^OE^ cells exposed to the AKT inhibitor MK-2206 showed suppressed AKT activation, enhanced AMPK activity, and decreased mTOR activity, along with increased epithelial protein (E-cadherin) and reduced mesenchymal marker (N-cadherin, Snail) expression (Fig. 5H). Furthermore, the AKT inhibition blocked the effects of MEDAG overexpression on breast cancer progression and EMT (Fig. 5I–K).

**Fig. 6:**
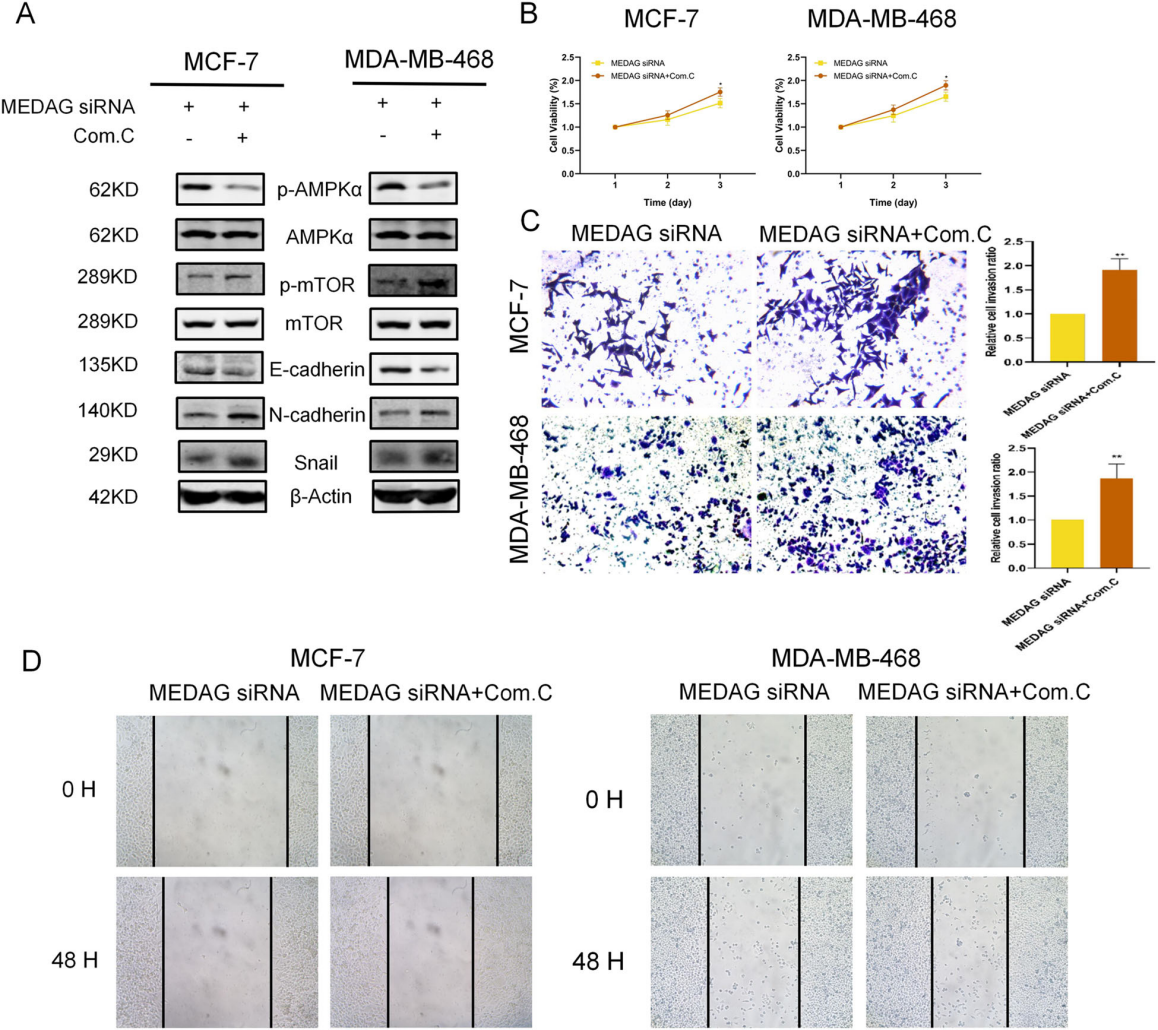
MEDAG regulates breast cancer progression and EMT via the AKT/AMPK/mTOR pathway. **A** Western blot analysis of p-AMPK, AMPK, p-mTOR, mTOR, and EMT protein expression in MEDAG^KD^ cells and MEDAG^KD^ cells treated with Compound C (10 µM, 24 h) in MCF-7 and MDA-MB-468 cell lines. **B** Cell growth was measured by CCK-8 in breast cancer cells treated as described above. **C** Transwell assay showing the cell invasion abilities in the groups treated as described above. Right: quantitative analysis of the invasion ratio is shown. **D** Wound-healing was used to examine the migration ability in two cell lines treated as described above. The values represent mean ± SD of three independent experiments. *P < 0.5, **P < 0.01 vs the corresponding group.

As shown in Fig. 6, the MEDAG^KD^ cells treated with Compound C, which is an inhibitor of AMPK activity, displayed suppressed AMPK activation and elevated p-mTOR protein levels with decreased E-cadherin and enhanced N-cadherin and Snail (Fig. 6A). Proliferation, invasion, and migration were promoted in the MEDAG^KD^ cells treated with Compound C (Fig. 6B–D). These results indicate that MEDAG promotes breast cancer progression and EMT by activating the AKT/AMPK/mTOR pathway.

### MEDAG reduces epirubicin sensitivity in breast cancer cells

**Fig. 7:**
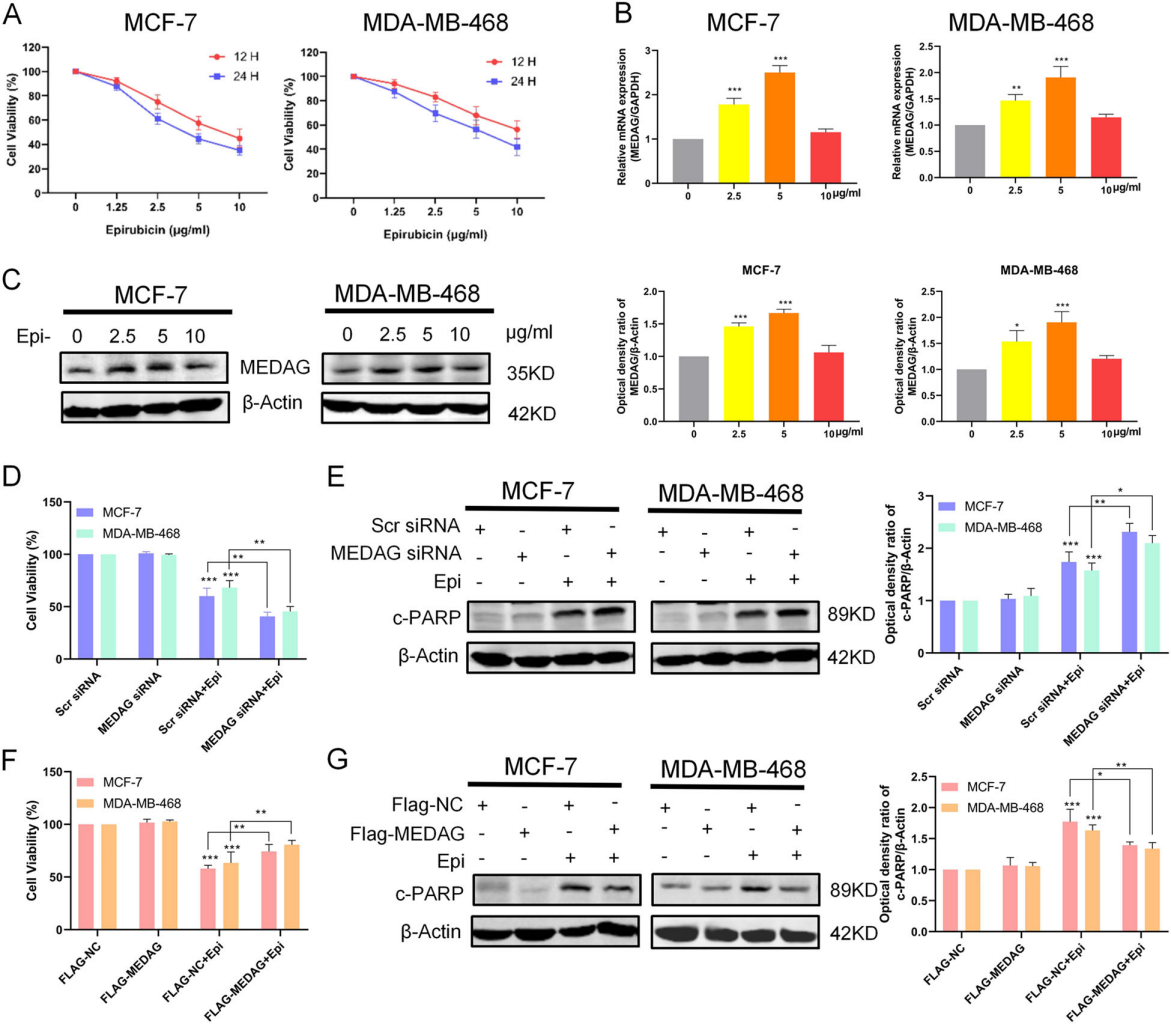
MEDAG reduces epirubicin sensitivity in breast cancer cells. **A** Cell proliferation was detected by CCK-8 after different concentrations (0, 1.25, 2.5, 5, or 10 µg/ml) and different durations (12 h or 24 h) of epirubicin treatment in two breast cancer cell lines. **B** mRNA level of MEDAG in breast cancer cells treated with different concentration of epirubicin (0, 2.5, 5, or 10 µg/ml) was detected by qRT-PCR. **C** Western blot analysis showing the protein expression of MEDAG in breast cancer treated as described above. Right: quantitative analysis of the optical density ratio of MEDAG compared with β-actin is shown. **D** Cell viability was assessed by CCK-8 after MEDAG knockdown and treatment with epirubicin (5 µg/ml,12 h). **E** The expression of PARP was detected by western blot analysis of cells treated as described above. Right: quantitative analysis of the optical density ratio of c-PARP compared with β-actin is shown. **F** Cell viability was assessed by CCK-8 after MEDAG overexpression and treatment with epirubicin. **G** The expression of PARP was detected by western blot analysis of cells treated as described above. Right: quantitative analysis of the optical density ratio of c-PARP compared with β-actin is shown. The values represent mean ± SD of three independent experiments. *P < 0.05, **P < 0.01, ***P < 0.001 vs the corresponding group.

MEDAG may contribute to the response to drugs according to the GO analysis (Fig. S1A), and our results suggest that MEDAG induces the EMT program, which is related to drug resistance^4^. Thus, we examined the relationship between MEDAG and drug effects. Epirubicin, which is a common chemotherapy agent, inhibits cell proliferation and promotes apoptosis in breast cancer^11^. As shown in Fig. 7A, MCF-7 and MDA-MB-468 cells were suppressed by epirubicin in a time- and concentration-dependent manner. To determine MEDAG expression in response to treatment with epirubicin, we treated MCF7 and MDA-MB-468 cells with different concentrations of epirubicin (0, 2.5, 5, or 10 µg/ml) for 12 h and observed that the MEDAG mRNA levels and protein levels were increased at higher concentrations (0–5 µg/ml) but returned to the control levels at 10 µg/ml in the two cell lines (Fig. 7B, C).

To verify the role of MEDAG in epirubicin-induced apoptosis, we used CCK-8 assays and western blot analyses to assess the proliferation and apoptosis of MCF-7 and MDA-MB-468 cells. The CCK-8 results showed that the cell viability of the MEDAG knockdown and control groups did not differ at 12 h, but MEDAG knockdown significantly promoted the inhibitory effect of epirubicin on the proliferation of breast cancer cells (Fig. 7D). To further examine whether MEDAG knockdown enhances chemotherapeutic sensitivity in MCF-7 and MDA-MB-468 cells, we detected the apoptosis-related protein PARP in the cells in response to epirubicin after silencing MEDAG. The results showed that MEDAG^KD^ enhanced the PARP protein levels when the cells were treated with 5.0 µg/ml epirubicin for 12 h compared to the treatment with the scramble siRNA cells and epirubicin (Fig. 7E). In contrast, MEDAG overexpression significantly reduced epirubicin sensitivity in breast cancer. The results showed that MEDAG^OE^ inhibited the effect of epirubicin on proliferation and decreased PARP expression in response to epirubicin (Fig. 7F, G). Moreover, MEDAG overexpression reduced epirubicin sensitivity in the MEDAG^KD^ cells (Fig. S5). Therefore, these results suggest that MEDAG has a protective effect on epirubicin-induced apoptosis in breast cancer cells.

### MEDAG regulates epirubicin-induced apoptosis through the AKT/AMPK pathway

**Fig. 8:**
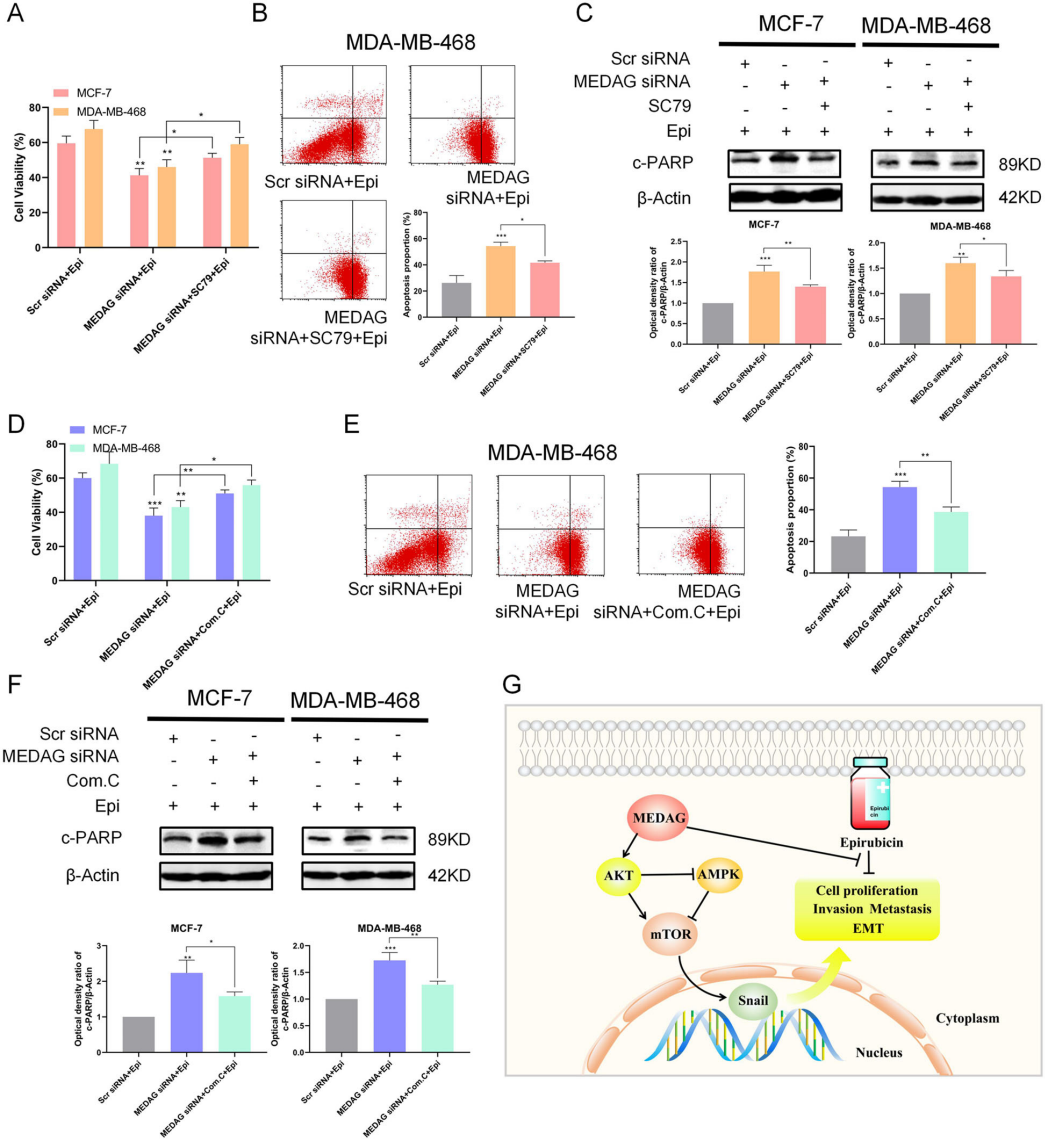
MEDAG regulates epirubicin-induced apoptosis through the AKT signaling. **A** Cells were transfected with scramble siRNA or MEDAG siRNA and then treated with epirubicin alone or with SC79 for 2h before the epirubicin treatment. Cell viability was assessed by CCK-8 in cells treated as described above. **B** Analysis of apoptosis with FACS in MDA-MB-468 cells treated as described above. Down: quantitative analysis of the apoptosis ratio. **C** The expression of c-PARP was detected by western blot analysis of breast cancer cells treated as described above. Down: quantitative analysis of the optical density ratio of c-PARP compared with β-actin is shown. **D**–**F** Cells were transfected with scramble siRNA or MEDAG siRNA and then treated with epirubicin alone or with Compound C for 24h before the epirubicin treatment. Cell viability was assessed by CCK-8 and analysis of apoptosis with FACS in MDA-MB-468 cells, and the expression of c-PARP was detected by western blot analysis of breast cancer cells treated as described above. Quantitative analyses of the apoptosis ratio and the optical density ratio of c-PARP compared with β-actin are shown. The values represent mean±SD of three independent experiments. *P < 0.05, **P < 0.01, ***P < 0.001 vs the corresponding group. **G** Proposed model of MEDAG-induced biological function in breast cancer cells.

Our previous studies investigated whether the AKT/AMPK pathway could be regulated by MEDAG; then, we examined whether this signaling pathway was involved in epirubicin-induced apoptosis. Fig. 8A–C shows that MEDAG^KD^ cells treated with SC79 and epirubicin had a higher cell viability, lower cell apoptosis, and a lower level of PARP expression than the MEDAG^KD^ cells treated with epirubicin alone. Next, we investigated cell proliferation and apoptosis in MEDAG^KD^ cells with or without Compound C. The CCK-8 assay showed that MEDAG^KD^ with Compound C also augmented cell proliferation following the treatment with epirubicin, while the FACS and western blot results showed that MEDAG^KD^ with Compound C inhibited apoptosis in response to epirubicin (Fig. 8D–F).

## Discussion

Breast cancer progression threatens the health of women worldwide and leads to poor prognoses. The identification of potential targets in cancer progression may help develop promising diagnostic and therapeutic management strategies. In this study, we identified the role of MEDAG in breast cancer and determined the possible signaling pathway involved. Our results showed that MEDAG was highly expressed in breast cancer and that breast cancer patients with high MEDAG showed aggressive vascular invasion, lymph node metastasis, and poor disease-free survival. The in vitro results verified that the knockdown of MEDAG inhibited proliferation, the pro-metastatic phenotype and EMT in breast cancer, and that the overexpression of MEDAG had the opposite effect. The in vivo experiments indicated that the knockdown of MEDAG suppressed tumor growth and metastasis. Then, the mechanistic experiments demonstrated that MEDAG regulated cancer progression and EMT through the AKT/AMPK/mTOR pathway. Regarding treatment, our study showed that MEDAG reduced epirubicin sensitivity in breast cancer cells and protected epirubicin-induced apoptosis through the AKT/AMPK pathway.

EMT characteristics, including invasion, dissemination, and metastasis, are altered during cancer progression^12,13^. Previous studies have demonstrated that before departure from the primary tumor, metastatic colonies usually undergo EMT and move to distant sites^14,15^. Furthermore, mouse experiments investigating breast cancer have indicated that the activation of the EMT program in primary breast tumors is pivotal for cancer cell dissemination to the lung^16,17^. In particular, the important event in EMT is the loss of E-cadherin, which could be directly repressed at the transcriptional level by the EMT-inducing transcription factor (EMT-TF) Snail, which is a zinc finger protein belonging to the Snail superfamily^18,19^. Additionally, Snail expression was closely associated with dedifferentiation and metastasis in patients with breast cancer^20^. On the one hand, we observed that silencing MEDAG tended to result in epithelial-like changes and that the overexpression of MEDAG resulted in a mesenchymal-like phenotype (decreased E-cadherin and, elevated N-cadherin and Snail). On the other hand, our study demonstrated that MEDAG overexpression was associated with increased vascular invasion and lymph node metastasis in patient samples, and MEDAG enhanced the pro-metastatic phenotype in breast cancer cell lines in vitro, along with increased metastasis outcome These results indicated that MEDAG regulated EMT to promote breast cancer invasion and metastasis.

Emerging evidence suggests that the Akt/AMPK/mTOR pathway is a crucial pathway for cell growth and metabolism^10^. The PI3K/Akt/mTOR pathway regulates protein synthesis, tumor growth, metastasis, EMT-TFs, and the EMT program^21–23^. AMPK acts opposite to AKT, and the mTOR pathway is negatively regulated by AMPK^24,25^. Our experiments indicated that MEDAG activated the AKT/AMPK/mTOR pathway to enhance proliferation, migration, invasion and EMT, and that an inhibitor or agonist could partially reverse the above progression, suggesting that MEDAG may influence breast cancer by regulating this pathway.

Epirubicin, which is among the most common drugs in cancer regimens, has been recommended as the first-line chemotherapy for patients with breast cancer. Epirubicin sensitivity in breast cancer cells determines the effectiveness of therapy. However, the underlying factors and mechanisms involved in chemotherapeutic sensitivity have not been elucidated. Previous studies have indicated that the activation of EMT is strongly correlated with drug resistance in multiple cancers^26,27^. Many studies have examined the responses to chemotherapy in patients with breast cancer and revealed the close association between an increased EMT-associated gene profile and treatment resistance, and the elevated expression level was partially caused by EMT activation in cancer cells^28^. As shown in our study, MEDAG promoted the EMT program. Moreover, increased chemosensitivity was observed in the MEDAG-silenced cancer cells, and MEDAG may modify the sensitivity of breast cancer cells to epirubicin by regulating the AKT/AMPK pathway.

One limitation of the present study is that this study lacks deeper mechanistic insight into how MEDAG regulates the AKT/AMPK/mTOR pathway. We speculate that AKT may be regulated by MEDAG in a direct or indirect manner. However, the detailed mechanism remains to be explored in future studies. Additionally, AMPK exhibits different functions towards AKT activities, which may be determined by distinct cellular and/or tissue contexts. In our study, we observed that silencing MEDAG decreased p-AKT activity and increased the p-AMPK protein level; a limitation is that this result may be cell-dependent, and we will continue to pay attention to this issue and perform further studies.

In conclusion, we show the role and corresponding mechanism of MEDAG in breast cancer for the first time. Our study reveals that MEDAG is highly expressed in breast cancer and is associated with poor survival. MEDAG promotes breast cancer cell proliferation, pro-metastasis, and EMT through the AKT/AMPK/mTOR pathway and reduces epirubicin sensitivity in breast cancer cells via AKT/AMPK.

## Supplementary information

Supplementary Figure legend.

Supplementary Figure 1.

Supplementary Figure 2.

Supplementary Figure 3.

Supplementary Figure 4.

Supplementary Figure 5.

Supplementary Table 1.
