# Supplementary material for: MEDAG enhances breast cancer progression and reduces epirubicin sensitivity through the AKT/AMPK/mTOR pathway
Source: Cell Death Dis. 2021 Jan 18;12(1):97. doi: 10.1038/s41419-020-03340-w (PMC7814033; doi:10.1038/s41419-020-03340-w)
Supplement: Supplementary file 1 — Supplementary Figure legend. [file 41419_2020_3340_MOESM1_ESM.docx]

**Supplementary figure legends**

**Supplementary Figure 1**. GO analysis of MEDAG by R software and DAVID analysis. (A) The biological process results showed that the DEGs were particularly enriched in cell adhesion, positive regulation of cell proliferation, angiogenesis and migration, response to drug, immune/inflammatory response, aging, etc. (B) The molecular function analysis showed that the DEGs were enriched in calcium ion binding, receptor activity, growth factor activity, etc. (C) The cellular component analysis showed that the DEGs were enriched in proteinaceous extracellular matrix, extracellular matrix, etc.

**Supplementary Figure 2**. (A) Top five predicted protein models of MEDAG. (B) Predicted ligand binding sites: binding residues are shown as blue ball and sticks. Predicted binding ligand is shown in green yellow sphere. Predicted ligands are as follows: NUCLEIC ACIDS (ligand binding site residues: 54, 73, 75, 96, 98, 99, 134, 138, 167, 169, 189, 193, 225, and 250), N,O6-DISULFO-GLUCOSAMINE (107 and 108), ALPHA-D-MANNOSE (76 and 99), (R)-((2R,3S,4R,5R,6R)-3-HYDROXY-2-(HYDROXYMETHYL)-5-((R)-3-HYDROXYTETRADECANAMIDO)-6-(PHOSPHONOOXY)TETRAHYDRO-2H-PYRAN-4-YL) 3-HYDROXYTETRADECANOATE (172 and 196), and ZINC 2+ (75 and 81).

**Supplementary Figure 3**. MEDAG overexpression enhanced cell proliferation and the pro-metastasis phenotype in breast cancer cells. (A-B) Overexpression efficiency of MEDAG in MCF-7 and MDA-MB-467 cell lines. (C) MEDAG overexpression enhanced breast cancer cell proliferation in MCF-7 (up) and MDA-MB-468 (down) cells. Cell proliferation was measured by a CCK-8 assay. (D) Transwell assays showing that MEDAG overexpression increased cell invasion in MCF-7 (up) and MDA-MB-468 (down) cells. Right: quantitative analysis of the invasion ratio is shown. (E) Wound healing assay showing that MEDAG overexpression enhanced cell migration. Representative images are shown at 0 h and 48 h. The values represent the mean±SD of three independent experiments. *p<0.5, **p<0.01 vs the control group.

**Supplementary Figure 4**. MEDAG overexpression rescues the inhibited cell proliferation and pro-metastasis phenotype in MEDAG^KD^ cells. (A) Transwell assays showing that MEDAG overexpression increased cell invasion in MEDAG^KD^ cells. Quantitative analysis of the invasion ratio is shown. (B) MEDAG overexpression rescued MEDAG^KD^ cells’ proliferation in MCF-7 (up) and MDA-MB-468 (down) cells. Cell proliferation was measured by a CCK-8 assay. (C) Wound healing assay showing that MEDAG overexpression in MEDAG^KD^ cells elevated MEDAG^KD^ cell migration. Representative images are shown at 0 h and 48 h. The values represent the mean±SD of three independent experiments. *p<0.05, **p<0.01, ***p<0.001, ****p<0.0001 vs the corresponding group.

**Supplementary Figure 5**. MEDAG overexpression reduces epirubicin sensitivity in MEDAG^KD^ cells. (A) Cell viability was assessed by CCK-8 after MEDAG overexpression in MEDAG^KD^ cells treated with or without epirubicin. (B) The expression of PARP was detected by a western blot analysis of cells treated as described above. Right: quantitative analysis of the optical density ratio of c-PARP compared with β-actin is shown. The values represent the mean±SD of three independent experiments. *p<0.05, **p<0.01, ***p<0.001 vs the corresponding group.
