# Supplementary figures and images for: MEDAG enhances breast cancer progression and reduces epirubicin sensitivity through the AKT/AMPK/mTOR pathway
Source: Cell Death Dis. 2021 Jan 18;12(1):97. doi: 10.1038/s41419-020-03340-w (PMC7814033; doi:10.1038/s41419-020-03340-w)

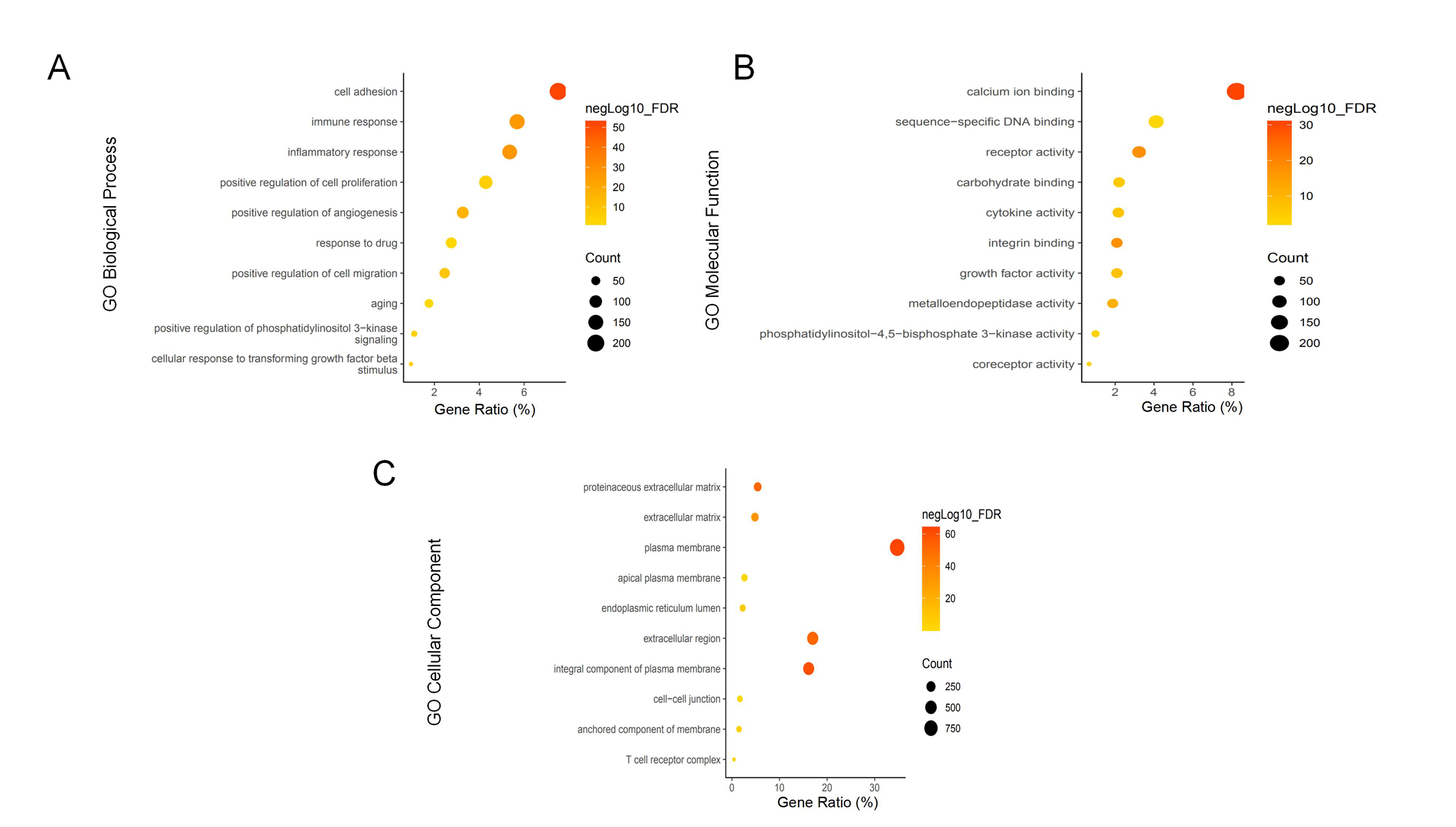

Supplement: Supplementary file 2 — Supplementary Figure 1. [file 41419_2020_3340_MOESM2_ESM.tif]

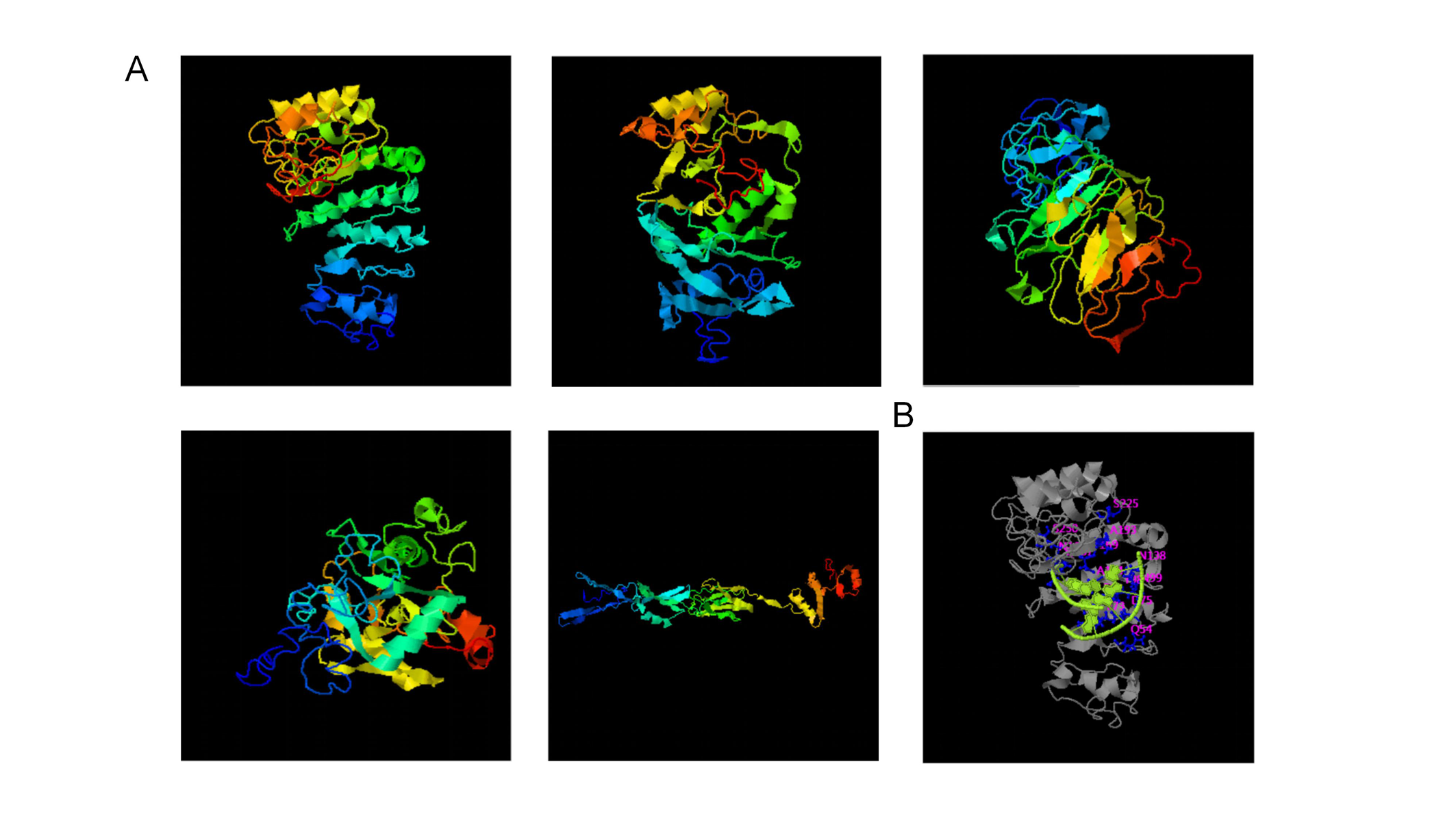

Supplement: Supplementary file 3 — Supplementary Figure 2. [file 41419_2020_3340_MOESM3_ESM.tif]

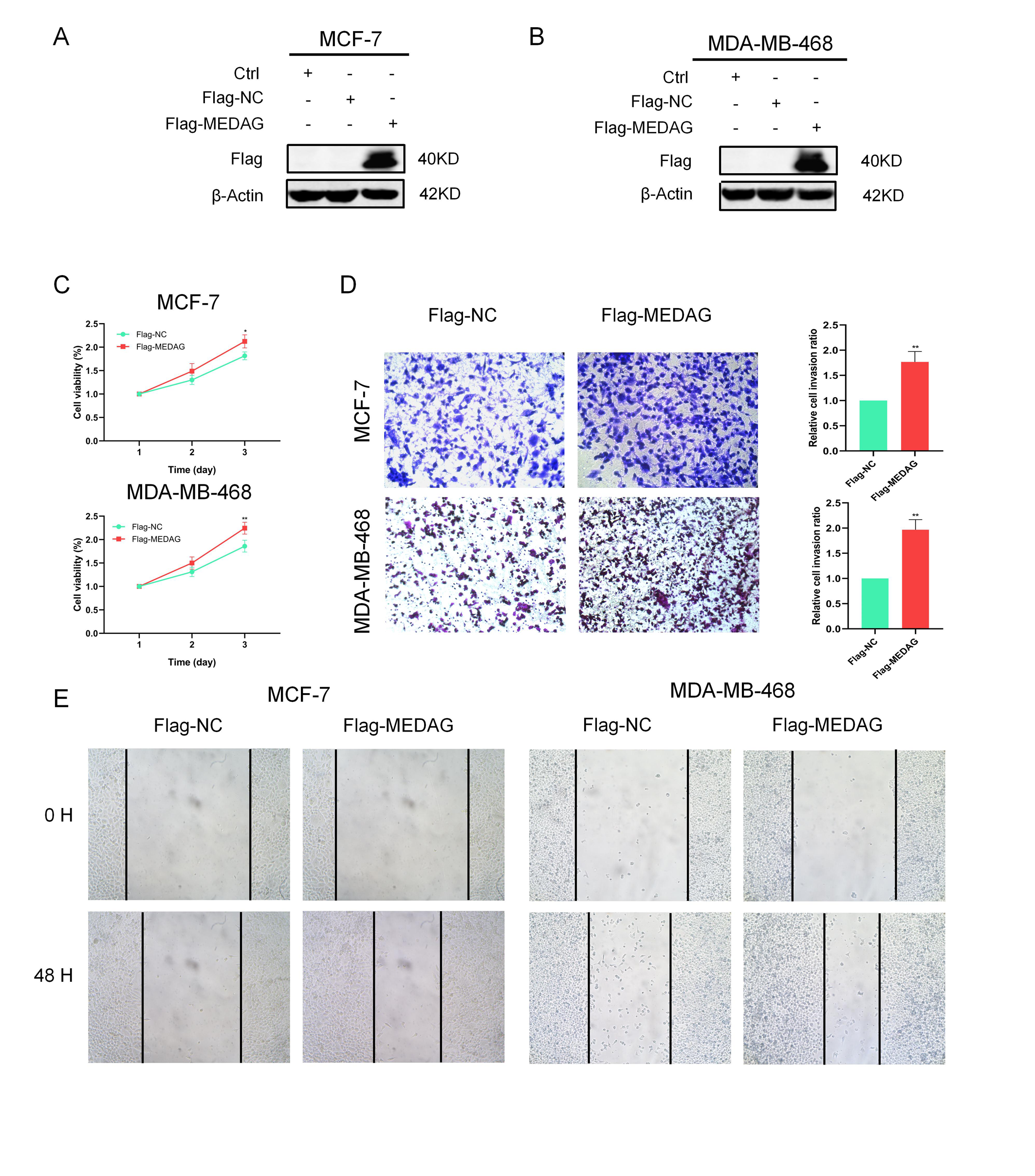

Supplement: Supplementary file 4 — Supplementary Figure 3. [file 41419_2020_3340_MOESM4_ESM.tif]

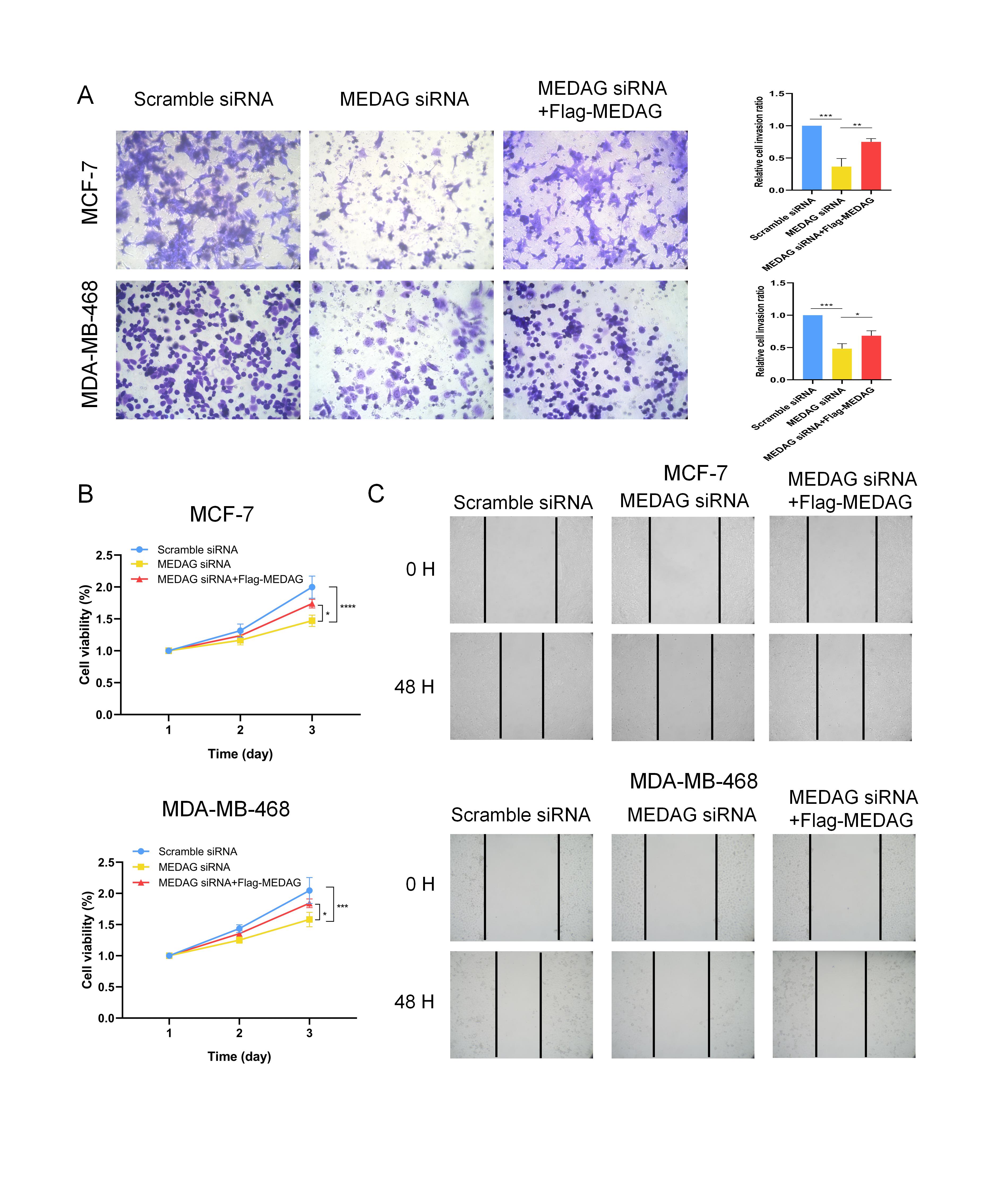

Supplement: Supplementary file 5 — Supplementary Figure 4. [file 41419_2020_3340_MOESM5_ESM.jpg]

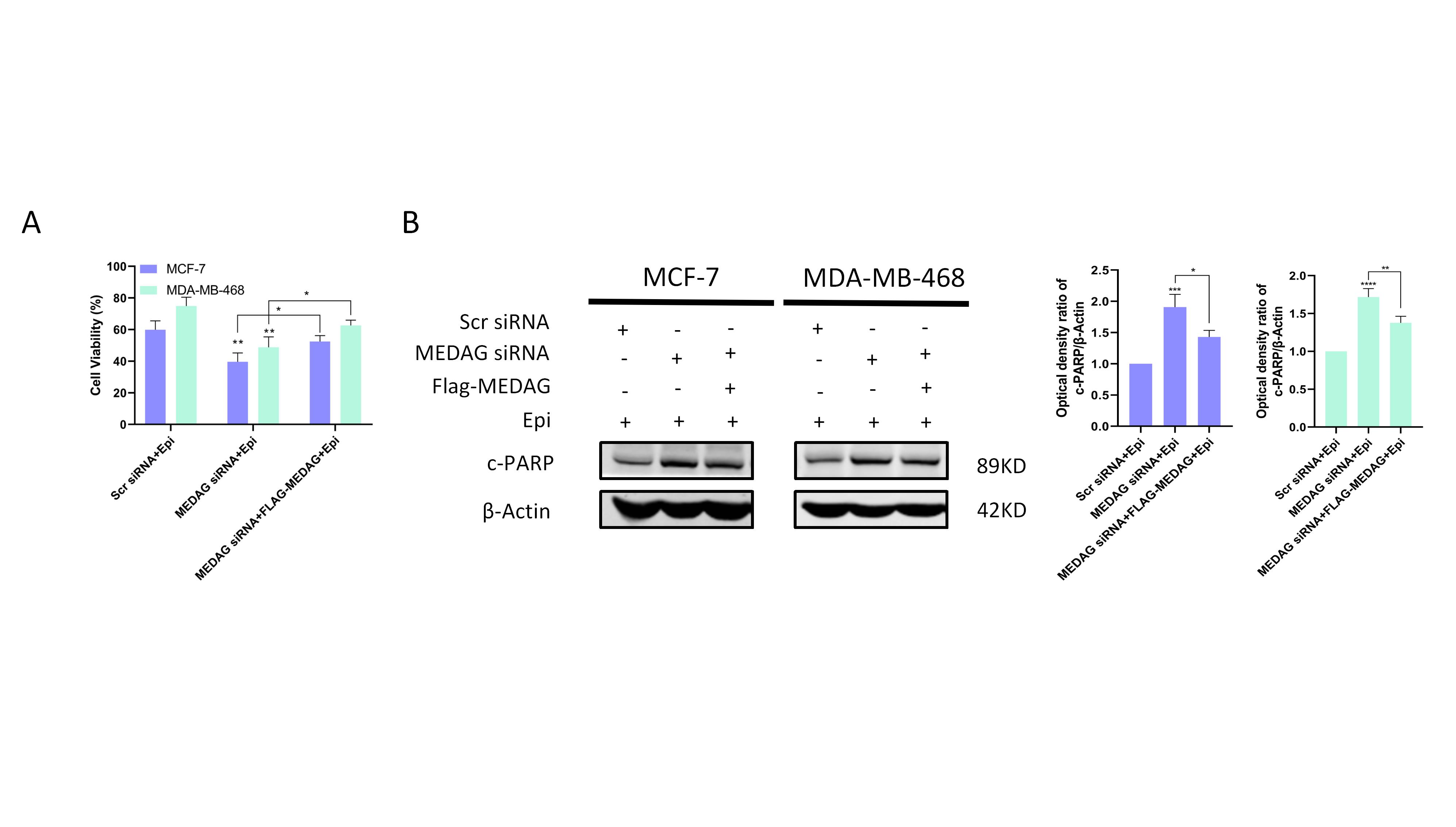

Supplement: Supplementary file 6 — Supplementary Figure 5. [file 41419_2020_3340_MOESM6_ESM.jpg]
