# Supplementary material for: MEDAG enhances breast cancer progression and reduces epirubicin sensitivity through the AKT/AMPK/mTOR pathway
Source: Cell Death Dis. 2021 Jan 18;12(1):97. doi: 10.1038/s41419-020-03340-w (PMC7814033; doi:10.1038/s41419-020-03340-w)
Supplement: Supplementary file 7 — Supplementary Table 1. [file 41419_2020_3340_MOESM7_ESM.docx]

**Supplementary Table 1. Primary antibodies and secondary antibodies for Western blotting**

| Antibody | Concentration | Article number | Company |
| --- | --- | --- | --- |
| MEDAG | 1:500 | orb326838 | Biorbyt |
| E-cadherin | 1:200 | sc-7870 | Santa Cruz |
| N-cadherin | 1：1000 | 13116 | Cell Signaling Technology |
| Snail | 1：1000 | 3879 | Cell Signaling Technology |
| p-Akt473 | 1：1000 | 4060 | Cell Signaling Technology |
| Akt | 1：1000 | 2920 | Cell Signaling Technology |
| p-AMPK | 1：1000 | 2535 | Cell Signaling Technology |
| AMPK | 1：1000 | 5831 | Cell Signaling Technology |
| p-mTOR | 1：1000 | 5536 | Cell Signaling Technology |
| mTOR | 1：1000 | 2983 | Cell Signaling Technology |
| FLAG | 1：5000 | F1804 | Sigma |
| PARP | 1:1000 | 9532 | Cell Signaling Technology |
| β-Actin | 1：10000 | A5441 | Sigma |
| Anti-rabbit IgG (H+L) | 1：10000 | 5151 | Cell Signaling Technology |
| Anti-mouse IgG (H+L) | 1：10000 | 5257 | Cell Signaling Technology |
